# Direct mail improves knowledge of basic life support guidelines in general practice: a randomised study

**DOI:** 10.1186/1757-7241-20-72

**Published:** 2012-10-14

**Authors:** Niels Secher, Mette Marie Mikkelsen, Kasper Adelborg, Ronni Mikkelsen, Erik Lerkevang Grove, Jens Mørch Rubak, Peter Vedsted, Bo Løfgren

**Affiliations:** 1 Department of Anaesthesiology, Aarhus University Hospital, Aarhus, Denmark; 2 Institute of Clinical Medicine, Aarhus University, Aarhus, Denmark; 3 Faculty of Health Sciences, Aarhus University, Aarhus, Denmark; 4 Department of Internal Medicine, Regional Hospital of Randers, Denmark; 5 Emergency Department, Aarhus University Hospital, Aarhus, Denmark; 6 Department of Cardiology, Aarhus University Hospital, Aarhus, Denmark; 7 Central Region Denmark, Viborg, Denmark; 8 Research Unit for General Practice, Aarhus University, Aarhus, Denmark; 9 Research Centre for Emergency Medicine, Aarhus University Hospital, Aarhus, Denmark

**Keywords:** Basic life support, First aid, Cardiopulmonary resuscitation (CPR), Education, Guidelines, General practice

## Abstract

**Background:**

Implementation of new guidelines into clinical practice is often incomplete. Direct mail is a simple way of providing information to physicians and may improve implementation of new guidelines on basic life support (BLS). The aim of this study was to describe knowledge of the most recent European Resuscitation Council (ERC) Guidelines for BLS among general practitioners (GPs) and investigate whether direct mail improves theoretical knowledge of these guidelines.

**Methods:**

All general practice clinics (n=351) in Central Denmark Region were randomised to receive either direct mail (intervention) or no direct mail (control). The direct mail consisted of the official ERC BLS/AED poster and a cover letter outlining changes in compression depth and frequency in the new guidelines. In general practice clinics randomised to intervention, every GP received a direct mail addressed personally to him/her. Two weeks later, a multiple-choice questionnaire on demographics and BLS guidelines were mailed to GPs in both groups.

**Results:**

In total, 830 GPs were included in this study (direct mail, n=408; control, n=422). The response rate was 58%. The majority (91%) of GPs receiving direct mail were familiar with BLS Guidelines 2010 compared to 72% in the control group (P<0.001). Direct mail improved knowledge of the new recommended chest compression depth (67% vs. 40%, P<0.001) and chest compression frequency (62% vs. 40%, P<0.001).

**Conclusion:**

Direct mail improved knowledge of changes in BLS guidelines and thus facilitated the implementation of this knowledge into clinical practice. Resuscitation councils and medical societies may consider using direct mail as a simple strategy to facilitate implementation of changes in clinical guidelines.

## Background

Cardiac arrest carries a poor prognosis, but prompt and effective basic life support (BLS) can double or triple survival [1]. The frequency of cardiac arrests treated by GPs has been reported to range from 15/100.000 to 77/100.000 patients [2,3]. Accordingly, GPs’ initiation of effective BLS may be an important factor in reducing mortality from cardiac arrest [4].

When treatment of a medical emergency is not performed on a daily basis, simple guidelines for optimal care are needed. There is a great willingness among GPs to perform BLS, but also a gap between actual BLS knowledge and international resuscitation guidelines [5]. Similarly, other studies have reported that the time needed to implement resuscitation guidelines in emergency medical services (EMS) is 1.5 years [6,7]. Much effort has been put into creating simple BLS guidelines, but if not implemented into clinical practice this work is of no use. Implementing new guidelines in clinical practise is challenging and it is important to know the effect of disseminating knowledge through different medias, e.g. a simple direct mailing strategy.

The aim of this study was to investigate the knowledge of the European Resuscitation Council (ERC) Guidelines for Resuscitation 2010 among GPs and to test whether direct mail improves theoretical knowledge of new BLS guidelines.

## Methods

### Participants and ethics

In January 2011, all 830 eligible GPs in Central Denmark Region, representing 25% of GPs in Denmark, were included in this study. In accordance with the Danish National Committee on Biomedical Research Ethics, no ethical review committee approval was required.

### Study design

GPs were randomised to receive either direct mail (intervention) or not (control). To avoid potential bias if GPs in the same clinic were allocated to the control and direct mailing group, the unit of randomisation was general practice clinics. General practice clinics were randomly assigned to the two groups based on random numbers generated in Microsoft Excel version 14.2.3 (Microsoft Denmark, Hellerup, Denmark).

The direct mail consisted of an envelope containing a cover letter outlining changes in BLS guidelines (compression depth and frequency) and a copy of the official ERC poster on how to perform BLS with the use of an automated external defibrillator (AED). The information was mailed via the national postal service. In general practice clinics randomised to intervention, every GP received the direct mail addressed personally to him/her. Two weeks later, a questionnaire on BLS guidelines, self-evaluated BLS skills, demographics, and experience with cardiac arrest was mailed to GPs in all general practice clinics. Non-responders received a reminder including an additional questionnaire after 20 days. Responders entered a draw to win a BLS course for the general practice clinic. Questionnaires could be answered online or mailed back to the research group as a hard copy. The questionnaire consisted of nine multiple-choice questions on theoretical BLS and AED knowledge. Each question had four possible answers and was assessed according to the ERC Guidelines for Resuscitation 2010. A five-point Likert scale, ranging from “1=strongly disagree” to “5=strongly agree”, was used to quantify self-evaluated BLS skills. Demographics collected from all participants included age, gender, year of board certification (Table 1). Questions on experience with cardiac arrest included previous BLS course, self-education, previous BLS performance and BLS equipment in the general practice clinic (Table 2).

**Table 1:** Demographics

	**Control (n= 252)**	**Direct-mail (n=216)**
Age (years)	53±8	53±8
Gender		
Female	45%	41%
Male	55%	60%
Board certified (year)	1994 [1987; 2004]	1993 [1986; 2000]

Age are presented as mean±SD, Board certification are presented as median and interquartile range.

**Table 2:** Education, experience and equipment in general practice clinics

	**Control (n=252)**	**Direct-mail (n=216)**	**P-value**
Familiar with the ERC Guidelines for Resuscitation 2010	176 (69.8%)	189 (87.5%)	<0.001
Stay updated on changes in BLS recommendations	199 (79.0%)	164 (75.9%)	0.44
BLS course within the last 5 years	186 (73.8%)	164 (75.9%)	0.67
Has performed resuscitation in their clinic	95 (37.7%)	89 (41.2%)	0.45
Number of resuscitation attempts performed by general practitioner in their clinic	2 [1; 2]	2 [1; 2]	0.81
Automated external defibrillator available in their clinic	92 (36.5%)	56 (25.9%)	0.02
Ventilation equipment available in their clinic	240 (95.2%)	208 (96.3%)	0.65

The table show counts and proportion of general practitioners with recent education, experience and access to equipment in their clinics. Number of resuscitation attempts is presented as median and interquartile range. BLS: Basic Life Support, ERC: European Resuscitation Council.

### Data analysis

Questionnaires received by mail, were manually entered into an online database with an error rate below 0.3% per questionnaire, evaluated by re-entering 10% of the questionnaires. A mean Likert-scale score was calculated for each question on self-evaluated skills.

### Statistics

Continuous variables are reported as mean±SD and categorical variables reported as n (%). Number of resuscitation attempts in general practice clinic and age is reported as median and interquartile range. Data were tested for normality using D’Agostino-Pearson test. Between-group comparisons were performed using Mann-Whitney’s and Fisher’s exact test as appropriate. P-values of 0.05 or less were considered statistically significant. Calculations were performed using GraphPad Prism version 5.01 (GraphPad Software, La Jolla, CA, USA).

## Results

**Figure 1:**
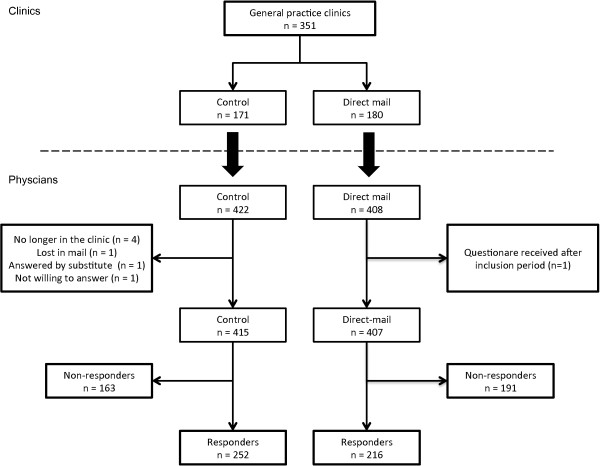
Flow-chart showing the randomisation and inclusion of study participants.

A total of 351 general practice clinics were included in the study (direct mail: n=180, control: n=171). This corresponded to a total of 830 GPs (direct mail: n=408, control: n=422). The response rate was 58%, 53% in the intervention group vs. 61% in the control group (Figure 1). There was no statistical difference in demographic characteristics between the two groups (Table 1).

The results from the theoretical BLS and AED questions are shown in Table 3. Direct mail improved knowledge of the new recommended chest compression depth (67% vs. 40%, P<0.001) and the new recommended chest compressions frequency (62% vs. 40%, P<0.001). In both groups, less than 30% knew the recommended primary diagnostic criteria for cardiac arrest. Instead of “unresponsive and abnormal or absent breathing”, more than 50% answered “unresponsive and no pulse”. To the question “a man has a cardiac arrest in your clinic, what is the first action you will take” more than 40% in both groups answered they would start BLS right away and less than 40% answered they would activate the EMS.

**Table 3:** Questions on basic life support and the use of an automated external defibrillator

	**Control (n=252)**	**Direct-mail (n=216)**	**P-value**
Diagnosis of a cardiac arrest	70 (27.7%)	58 (26.8%)	0.84
The first action to take when a person has cardiac arrest in your clinic	79 (31.3%)	73 (33.7%)	0.62
Recommended compression depth	100 (39.5%)	143 (66.2%)	<0.001
Recommended compression frequency	95 (37.7%)	133 (61.6%)	<0.001
Recommended compression ventilation ratio	176 (69.8%)	162 (75.0%)	0.25
Recommended volume for ventilation	231 (91.7%)	190 (88.0%)	0.22
When to place a person in the recovery position	240 (95.2%)	200 (92.6%)	0.25
When to use an automated external defibrillator at a cardiac arrest	220 (87.3%)	184 (85.2%)	0.67
How to place the automated external defibrillator pads	224 (88.9%)	183 (84.7%)	0.22

The table shows the questions asked in the questionnaire on basic life support and use of an automated external defibrillator. Correct answers to questions in the control and intervention group are presented as counts and proportion.

As shown in Table 4, the majority of GPs reported they felt confident in their ability to perform BLS and handling a person in cardiac arrests in their clinic. More than 55% thought more BLS training was needed among GPs. Up to 80% of GPs were making an effort to stay updated on changes in BLS recommendations. Information mainly originated from national journals for GPs or continuing education and to a lesser extent via the Internet.

**Table 4:** Self-evaluated skills

	**Control (n = 252)**	**Direct-mail (n = 216)**	**P-value**
“I feel confident performing BLS”	4.4±0.6 94.4%	4.4±0.6 95.8%	0.81
“I feel I can handle a person with cardiac arrest in my clinic”	4.1±0.7 87.7%	4.2±0.7 88.8%	0.38
“I think more BLS training relevant for general physicians are needed”	3.5±1.1 55.4%	3.6±1.0 59.7%	0.50

The table shows the questions asked in the questionnaire on self-evaluated skills. Answers are presented as mean Likert score±SD and proportion (%) stating “agree” or “strongly agree”. BLS: Basic Life Support.

Approximately 40% had performed BLS in their clinic with a median number of attempts of 2 [1; 2] and 2 [1; 2] respectively. The majority (90.4%) of GPs receiving direct mail stated familiarity with the ERC Guidelines for Resuscitation 2010 compared to 71.8% in the control group (P<0.001). Almost all general practice clinics had ventilation equipment available, whereas approximately 1/3 had access to an AED (Table 2).

## Discussion

This study demonstrates that direct mail significantly improves the knowledge of specific changes in BLS guidelines. However, the study also demonstrates room for improvement in the knowledge of the diagnosis of cardiac arrest and activation of the EMS.

Direct mail is a simple way of disseminating information to a large group of individuals. The significant improvement in knowledge of guidelines caused by this simple intervention makes this a favourable way of drawing attention to changes in guidelines. The improvement was restricted to the changes outlined in the personal cover letter (compression depth and frequency), whereas the poster outlining the entire BLS sequence did not seem to have an effect. This finding indicates that pushing information to GPs should be personally directed, easy to assess and preferably not in the form as a poster.

Compression depth and frequency have been changed in the ERC Guidelines for Resuscitation 2010 due to the importance of chest compressions in improving survival [1,8]. Even though direct mail significantly improved knowledge of compression depth and frequency there is nevertheless room for improvement since 1/3 of the direct mail group did not know the correct answer. This is in accordance with the physicians’ self-evaluated need for relevant BLS training among GPs.

The lack of knowledge of current guidelines was even more pronounced when it came to diagnosing cardiac arrest. Checking for carotid pulse is an inaccurate way of diagnosing cardiac arrest and is not recommended [9-11]. We found that more than 50% in both groups would check for pulse as a diagnostic criteria for cardiac arrest and not evaluate whether breathing is abnormal or absent. This diagnostic delay may result in loss of valuable time before initiation of BLS. Likewise, no less than 40% of GPs would initiate BLS instead of calling the EMS as the first action after diagnosing cardiac arrest. The importance of early activation of the EMS among GPs needs to be emphasised although the response to a questionnaire and what the physician would do in real life may differ. In almost all general practice clinics, the physician is not alone, and a secretary or a nurse would possibly immediately activate the EMS.

Previous studies have shown a discrepancy between self-evaluated compliance with guidelines and clinical practice [12]. Similar, in our study a high proportion of physicians stated familiarity with the ERC Guidelines for Resuscitation 2010 even though many answers were consistent with 2005 or even 2000 guidelines.

Another important finding of this study is that many GPs eventually will need to manage cardiac arrest in their clinic. Even though cardiac arrest is a rare event, 40% had performed an average of 2 BLS attempts in their clinic, making GPs an important factor in the chain of survival. It is well documented that BLS increases chances of successful defibrillation and increases survival to discharge [13]. Because 2/3 of GPs do not have access to an AED, BLS is their only treatment option during a cardiac arrest until the EMS arrives. Accordingly, it is crucial that new resuscitation guidelines are implemented in general practice.

Ideally GPs should attend a BLS/AED refresher course every two years and when new resuscitation guidelines are published [14]. Unfortunately time and money may be a considerably barrier for GPs to attend a hands-on training course and alternative training methods are needed. E-learning is suitable for reaching small groups over large distances, however the limited studies on this topic show conflicting results [15,16]. Retraining using a poster and a manikin may be a cost effective way of introducing skills and refresher training in general practice, but the beneficial effect must be evaluated in further studies [17,18]. Direct mail is suitable for drawing attention to changes, but the optimal method to improve general knowledge and skills on resuscitation guidelines is still to be found.

### Implications

Cardiac arrest carries a dismal prognosis and is frequently encountered in general practice, although rarely by the individual GP. Our findings call for a better implementation of guidelines in clinical practice. Furthermore, this study presents direct mail as a way to facilitate the implementation of guidelines to GPs.

### Limitations

This study tested the theoretical knowledge of BLS guidelines and not practical skills. Theoretically, BLS knowledge may not convert to practical skills. Studies on laypeople have however shown that training increases willingness to perform BLS [19]. Despite a follow up letter to non-responders the overall response rate was 58%, which may introduce selection bias. However, the controlled design with cluster-randomisation of the GPs and a non-differential selection bias makes the comparisons between the groups valid. If the responders were those most interested in the area and thus, with the highest knowledge, we may have overestimated the proportions with specific knowledge. Finally, based on this study it is not possible to elucidate the long-term implementation in the direct mail group as the assessment was made two weeks after mailing the cover letter and poster.

## Conclusion

Direct mail improves knowledge of changes in BLS guidelines and thus helps implement new guidelines in general practice. Still, measures to facilitate implementation of guidelines are warranted. Resuscitation councils and medical societies for general practice may consider using direct mail as a strategy for implementing changes in clinical guidelines.

## Abbreviations

BLS: Basic life support; GPs: General practitioners; EMS: Emergency medical services; ERC: European Resuscitation Council; AED: Automated external defibrillator.

## Competing interest

The authors declare that they have no competing interests.

## Authors’ contributions

NS, BL and KA contributed to design of the study, mailing and collecting questionnaires, data interpretation, statistical analysis, and drafting the manuscript. MM and RM participated in mailing and collecting questionnaires, data interpretation and drafting the manuscript. EG and PV contributed to design of the study, interpretation of the data and drafting the manuscript. JR provided contact to study participants, contributed to preparation of the questionnaire and drafting of the manuscript. All authors read and approved the final manuscript.
